# Optimal, Interpretable, and Personalized Immune Checkpoint Inhibitor Duration in Non‐Small Cell Lung Cancer: A Multicenter Real‐World Study

**DOI:** 10.1002/mco2.71032

**Published:** 2026-09-29

**Authors:** Siqi Wang, Jianhua Liu, Qiang Nie, Ruichuan Shi, Nan Xu, Qianqian He, Guanchao Ye, Jingyi Liu, Qiuyang Hou, Kexue Deng, Lu Wang, Taixue An, Anyi An, Xiujuan Qu, Jiangdian Song

**Affiliations:** ^1^ School of Health Management China Medical University Shenyang Liaoning China; ^2^ Department of Oncology Guangdong Provincial People's Hospital (Guangdong Academy of Medical Sciences) Southern Medical University Guangzhou China; ^3^ Department of Pulmonary Surgery Guangdong Lung Cancer Institute Guangdong Provincial People's Hospital (Guangdong Academy of Medical Sciences) Southern Medical University Guangzhou China; ^4^ Department of Medical Oncology The First Hospital of China Medical University Shenyang Liaoning Province China; ^5^ Department of Thoracic Surgery The First Affiliated Hospital of Zhengzhou University Zhengzhou Henan China; ^6^ The First Hospital of China Medical University Shenyang Liaoning China; ^7^ Division of Life Sciences and Medicine Department of Radiology The First Affiliated Hospital of University of Science and Technology of China (USTC) Hefei Anhui China; ^8^ Shengjing Hospital of China Medical University Shenyang Liaoning China; ^9^ Department of Laboratory Medicine Nanfang Hospital Southern Medical University Guangzhou China; ^10^ Jinzhou Medical University Jinzhou Liaoning Province China

**Keywords:** decision‐making, immunotherapy, non‐small cell lung cancer, prognosis, treatment duration

## Abstract

The optimal treatment duration of immune checkpoint inhibitors (ICIs) in non‐small cell lung cancer (NSCLC) remains undefined. We conducted a multicenter retrospective cohort study of 1054 patients with NSCLC treated with ICIs across six hospitals in China and one in the United States. Using a training cohort of 372 patients, we constructed a counterfactual random forest reward‐estimation matrix incorporating age, tumor diameter, and derived neutrophil‐to‐lymphocyte ratio, and an Optimal Policy Survival Tree (OPST) was developed to characterize phenotype‐specific associations between baseline clinical features, treatment duration patterns (short‐, intermediate‐, and long‐course), and survival outcomes. Two external cohorts (*n* = 324 and *n* = 358) were used for validation. The OPST reproducibly defined seven patient subgroups across datasets. Subgroups 4 and 6 (37.8%) showed more favorable overall survival (OS) associations with short‐course therapy (median OS 28.6 and 24.5 months), whereas Subgroups 3 and 5 (41.5%) showed more favorable associations with long‐course therapy (median OS 27.0 and 22.0 months; all *p* < 0.001). The OPST framework identified a subset of patients (18.1%) potentially associated with limited incremental survival benefit from prolonged treatment exposure. This transparent and interpretable framework based on routine clinical data may help inform future prospective evaluation of individualized ICI duration strategies, with the potential to support individualized treatment planning while reducing unnecessary treatment exposure.

## Graphical abstract

This multicenter retrospective study of 1054 patients with NSCLC developed an interpretable Optimal Policy Survival Tree using a counterfactual random forest reward‐estimation matrix based on age, tumor diameter, and dNLR. The model identified seven patient subgroups with distinct phenotype‐associated treatment‐duration patterns: 37.8% showed more favorable survival associations with short‐course ICI therapy, whereas 41.5% showed more favorable associations with long‐course therapy. These findings suggest that the framework may help inform future prospective evaluation of individualized ICI duration strategies while reducing unnecessary treatment exposure.

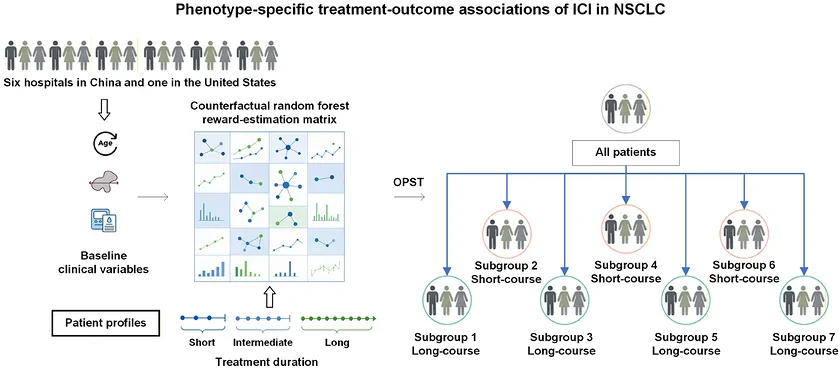

## 1 Introduction

Non‐small cell lung cancer (NSCLC) remains the leading cause of cancer‐related mortality worldwide, and despite advances in molecular diagnostics and targeted therapies, a substantial proportion of patients are diagnosed at advanced stages and rely on systemic treatments such as immunotherapy [1]. Immune checkpoint inhibitors (ICIs) that block PD‐1 or its ligand PD‐L1 have transformed the treatment landscape for NSCLC  [1, 2]. Contemporary management integrates histopathological classification, molecular profiling, and immune biomarkers to guide treatment selection, yet survival outcomes remain highly heterogeneous. Elevated PD‐L1 expression or a high tumor mutation burden (TMB) does not consistently result in longer overall survival (OS), and some patients with negative biomarkers derive long‐term survival benefit [3, 4, 5, 6]. These discrepancies highlight the need for robust prognostic models that complement existing biomarkers and guide personalized ICI therapy in patients with NSCLC.

In addition, the optimal duration of ICI therapy for individual patients with NSCLC is unclear. Although current guidelines cap treatment at 35 cycles, pivotal trials such as KEYNOTE‐024, KEYNOTE‐189, and IMpower010 report median exposures of only 10–16 cycles [7, 8, 9]. In routine practice, cycle counts vary even more because of early discontinuation driven by tumor response, toxicity, cost, and therapy switching [10, 11]. Although prolonged exposure may prolong progression‐free survival (PFS), unnecessary continuation can increase immune‐related toxicities without improving OS [5, 12, 13]. These results highlight the need for individualized treatment planning to optimize patient outcomes. In addition to pathology‐based biomarkers, baseline clinical variables, including age, longest tumor diameter (LD), Eastern Cooperative Oncology Group (ECOG) performance status, and the derived neutrophil‐to‐lymphocyte ratio (dNLR), have demonstrated prognostic value and could refine decisions regarding the duration of treatment [8, 9, 14, 15, 16].

Recent advances in artificial intelligence (AI) offer a path to predict ICI therapy outcomes before treatment begins [17]. However, most existing models operate as “black boxes,” providing limited insight into which patients are most likely to benefit, the optimal duration of treatment, and the associated survival gains that can be expected. Moreover, few approaches explicitly link baseline patient characteristics to treatment duration strategies, limiting their clinical applicability in guiding longitudinal therapeutic decisions. To address this gap, we developed an easily interpretable AI framework trained on real‐world data from six Chinese cancer centers and the Memorial Sloan Kettering Cancer Center (MSKCC) in New York City in the United States. The aim was to develop a clinician‐friendly, visually intuitive tool to enable personalized selection of ICI candidates, guide treatment duration, and predict OS.

In this study, we developed and externally validated an interpretable Optimal Policy Survival Tree (OPST) to stratify patients with NSCLC into clinically meaningful subgroups and identify phenotype‐specific optimal ICI treatment durations. We show that OPST consistently partitions patients into seven subgroups with distinct clinicopathological features and treatment–outcome associations, enabling identification of populations that benefit from short‐course versus extended therapy. These findings provide a transparent and clinically interpretable framework that may support individualized immunotherapy duration assessment and future hypothesis‐driven clinical investigation.

## 2 Results

### 2.1 Patient Cohorts

A total of 1054 patients were enrolled in this study following an initial screening of 2004 patients. The Guangdong Provincial People's Hospital contributed the training cohort (*n* = 372), and the two external validation cohorts comprised 324 and 358 patients (Figure 1 and Table 1), respectively. Overall, the median number of ICI therapy cycles was 5 (interquartile range [IQR] 2–9), the median follow‐up was 21.5 months (IQR 10.6–35.5 months), and median OS was 15.8 months (IQR 8.0–29.1 months), with 151 patients censored. Among 467 patients with early‐stage or locally advanced disease, the median number of combination‐therapy cycles was 4 (IQR 2–9). Among the remaining 484 patients with metastatic NSCLC, the median number of cycles was 5 (IQR 3–9). Information on the clinical stage was not available for patients in the MSKCC center.

**FIGURE 1:**
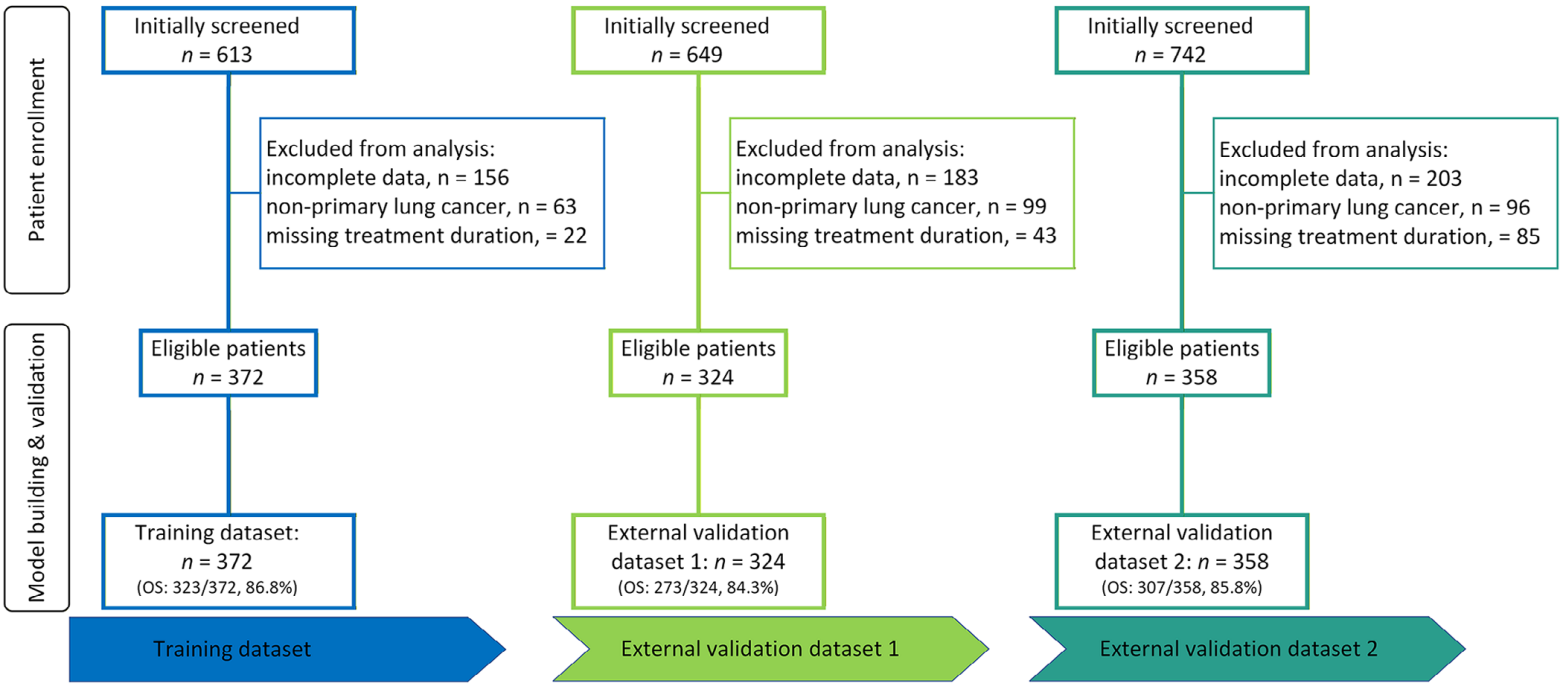
Patient enrollment and the overall survival optimal policy tree building and validation flowchart of this study.

**TABLE 1:** Patient characteristics in the training and two external validation datasets.

	Training (*n* = 372)	External validation 1 (*n* = 324)	External validation 2 (*n* = 358)
**Age**, year (median, IQR)	61 (55–68)	67 (61–72)	64 (57–71)
**Sex**			
Male (%)	304 (81.7)	248 (76.5)	222 (62.0)
Female (%)	68 (18.3)	76 (23.5)	83 (38.0)
**LD**, mm (median, IQR)	41 (25–60)	47 (32–65)	36 (23–60)
**Immunotherapy course**			
1–3 cycle (%)	157 (42.2)	131 (40.4)	148 (41.3)
4–6 cycle (%)	108 (29.0)	112 (34.6)	84 (23.4)
>6 cycle (%)	107 (28.8)	81 (25.0)	126 (35.3)
**Clinical stage**			
I‐III (%)	197 (53.0)	109 (33.6)	161 (44.9)
IV (%)	175 (47.0)	215 (66.4)	94 (26.3)
**Pathology subtype**			
LUAD (%)	189 (50.8)	178 (54.9)	187 (52.2)
LUSC (%)	87 (23.4)	136 (42.0)	99 (27.6)
Others (%)	96 (25.8)	10 (3.1)	72 (20.2)
**Smoking status**			
Yes (%)	254 (68.3)	181 (55.9)	205 (54.5)
No (%)	118 (31.7)	143 (44.1)	153 (45.5)
**dNLR** (median, IQR)	2.1 (1.5–3.1)	3.7 (2.8–5.5)	2.5 (1.8–4.2)
**OS**, month (median, IQR)	19.9 (11.2–40.3)	15.6 (8.1–24.1)	11.7 (4.3–25.5)

*Note*: Data on clinical stage were not available in the original MSKCC dataset, and data on sex were not available for 53 patients in the original MSKCC dataset.

Abbreviations: dNLR, pre‐therapy derived neutrophil‐to‐lymphocyte ratio; IQR, interquartile range; LD, longest diameter; LUAD, lung adenocarcinoma; LUSC, Lung squamous cell carcinoma; OS, overall survival; Sqc, Squamous cell carcinoma.

### 2.2 OPST‐Defined Subgroups

The OPST model was developed to explore phenotype‐specific associations between baseline characteristics, treatment duration patterns, and OS outcomes. Using a reward‐estimation matrix derived from the counterfactual random forest, the OPST partitioned the population into seven terminal subgroups on the basis of baseline dNLR, maximum tumor diameter (LD), and age (Figure 2 and Table 2). In the training cohort, long‐course therapy conferred a significant OS advantage in Subgroups 3 and 5 (both *p* < 0.05; Table 3). However, in Subgroups 1 and 7, OS did not differ significantly according to the ICI treatment duration (*p* =  0.27 and *p*  =  0.14 in Figure 3), and long‐course therapy did not outperform short‐course therapy.

**FIGURE 2:**
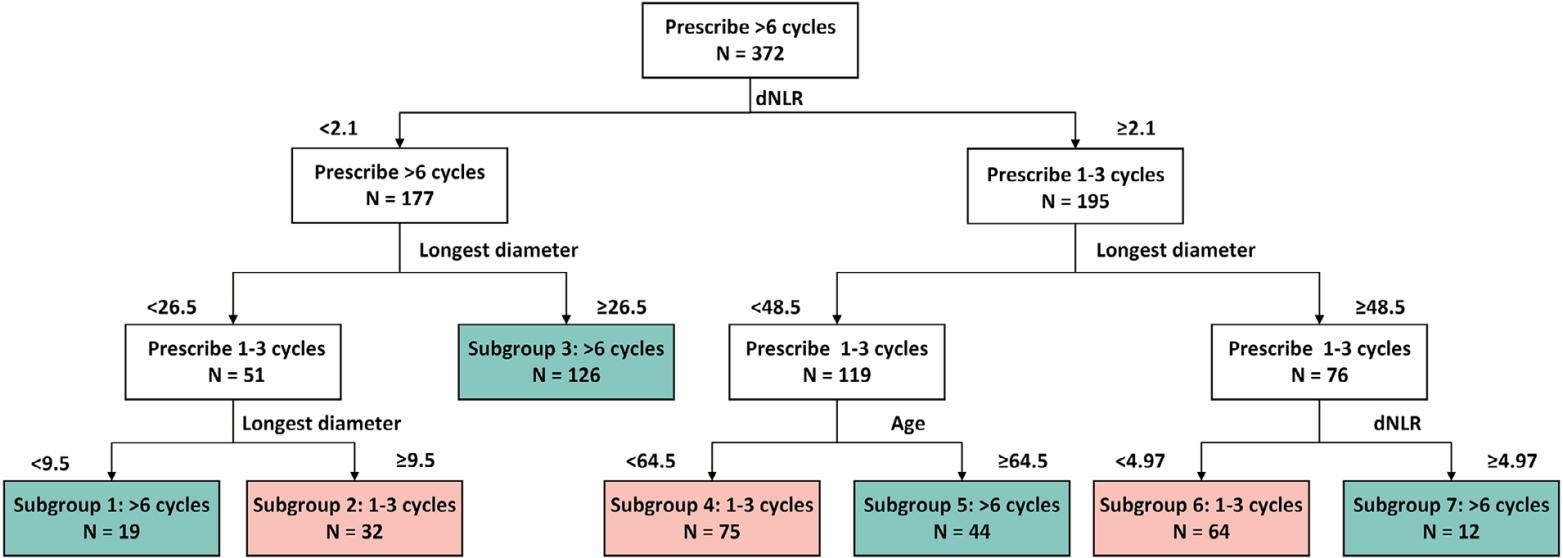
Overall Survival Optimal Policy Tree for duration of immune checkpoint inhibitor therapy for patients with non‐small cell lung cancer. Subgroup 1 included patients with lower dNLR (<2.1) and smaller longest diameter (LD < 9.5 mm). Subgroup 2 included patients with lower dNLR (<2.1) and intermediate tumors (9.5 ≤ LD < 26.5 mm). Subgroup 3 included patients with lower dNLR (<2.1) and longer LD (LD > 26.5 mm). Subgroup 4 included patients with higher dNLR (≥2.1) and LD <48.5 mm and age <64.5 years. Subgroup 5 included patients with higher dNLR (≥2.1) and LD <48.5 mm and age ≥64.5 years. Subgroup 6 included patients with 2.1 ≤ dNLR < 4.97 and LD ≥48.5 mm. Subgroup 7 included patients with dNLR ≥4.97 and LD ≥48.5 mm. dNLR, pre‐therapy derived neutrophil‐to‐lymphocyte ratio; LD. maximum tumor diameter.

**TABLE 2:** The definition, number of patients, and OPST‐recommended duration of seven subgroups by the Optimal Policy Survival Tree (OPST).

Subgroup	dNLR	LD (mm)	Age (year)	Training	External validation 1	External validation 2	OPST‐recommended treatment duration
1	< 2.10	< 9.5	—	19	4	9	Long
2	< 2.10	9.5–26.5	—	32	15	43	Short
3	< 2.10	≥ 26.5	—	126	44	66	Long
4	≥ 2.10	≥ 48.5	< 64.5	75	48	70	Short
5	≥ 2.10	< 48.5	≥ 64.5	44	87	70	Long
6	2.10–4.97	≥ 48.5	—	64	74	68	Short
7	≥ 4.97	≥ 48.5	—	12	52	32	Long

*Note*: Long: >6 cycles immunotherapy; Short: 1–3 cycles of immunotherapy; “—” represents no restriction. dNLR, pre‐therapy derived neutrophil‐to‐lymphocyte ratio; LD, longest diameter; OPST, optimal policy survival tree.

**TABLE 3:** Comparative analysis of overall survival in the four OPST‐defined subgroups associated with long‐course immunotherapy. *p* value was calculated by log‐rank test with Benjamini–Hochberg correction.

	Subgroup 1			Subgroup 3			Subgroup 5			Subgroup 7		
	*N*	Median (IQR)	*p*	N	Median (IQR)	*p*	*N*	Median (IQR)	*p*	N	Median (IQR)	*p*
**Training dataset**												
1–3 cycles	8	14.7 (10.2, 18.0)	0.250	54	20.9 (10.9, 30.5)	0.009	20	12.3 (9.0, 23.9)	0.045	6	16.7 (11.6, 18.4)	0.130
4–6 cycles	7	11.4 (11.1, 12.1)	0.250	34	14.3 (9.8, 30.1)	0.005	12	12.6 (6.3, 31.7)	0.033	2	25.5 (21.5, 29.4)	0.043
> 6 cycles	4	12.4 (11.7, 13.2)	Ref	38	33.0 (19.0, 68.5)	Ref	12	33.4 (20.7, 51.8)	Ref	4	70.3 (53.6, 84.1)	Ref
**External validation dataset 1**												
1–3 cycles	1	11.2 (11.2, 11.2)	0.47	12	6.0 (4.4, 8.0)	< 0.001	36	13.9 (6.5, 25.6)	0.018	28	8.1 (5.7, 16.3)	0.380
4–6 cycles	1	20.6 (20.6, 20.6)	1.00	20	7.0 (5.8, 17.8)	0.005	30	12.1 (9.9, 22.7)	0.014	17	17.5 (11.0, 24.1)	0.380
> 6 cycles	2	25.8 (24.6, 26.9)	Ref	12	25.0 (20.3, 36.3)	Ref	21	20.6 (17.3, 36.3)	Ref	7	23.1 (15.7, 28.1)	Ref
**External validation dataset 2**												
1–3 cycles	1	6.8 (6.8, 6.8)	0.076	23	5.5 (2.3, 8.0)	< 0.001	34	3.1 (1.8, 6.7)	< 0.001	13	6.1 (2.1, 10.2)	0.001
4–6 cycles	3	34.1 (22.5, 36.9)	0.994	15	9.1 (6.4, 13.9)	< 0.001	16	7.6 (6.7, 17.7)	0.006	6	19.6 (9.4, 25.7)	0.172
> 6 cycles	5	23.7 (19.3, 45.1)	Ref	28	27.5 (14.7, 42.5)	Ref	20	18.7 (14.7, 34.7)	Ref	13	26.5 (21.1, 41.2)	Ref

**FIGURE 3:**
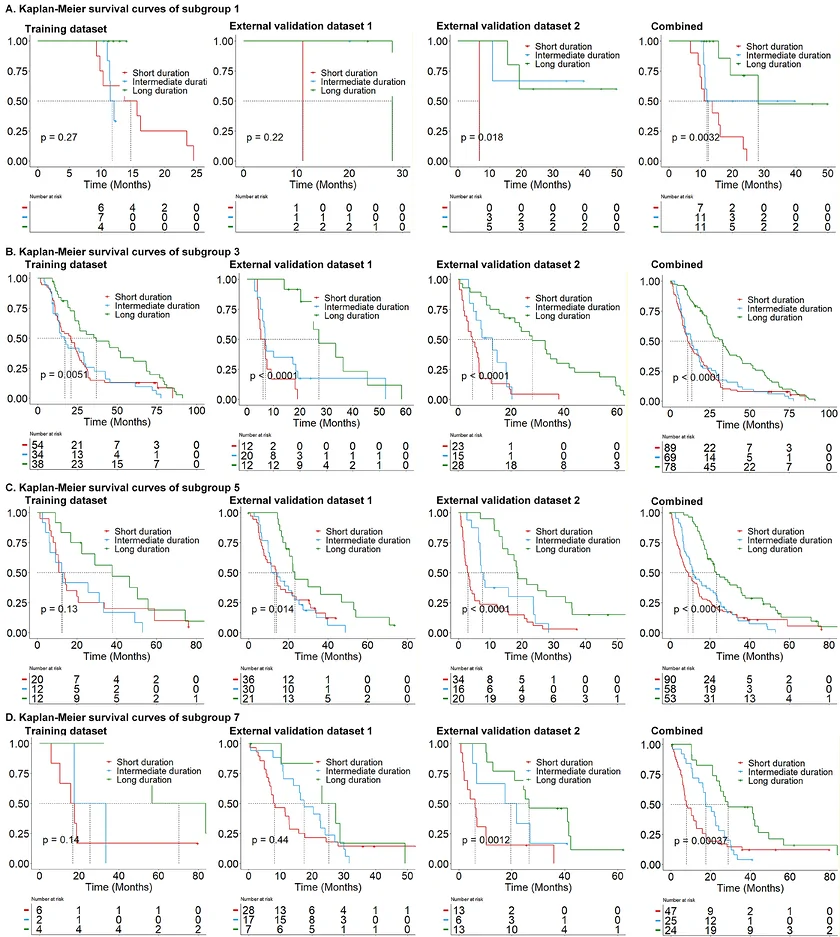
Kaplan–Meier overall survival curves for Optimal Policy Survival Tree (OPST)‐defined subgroups associated with long‐course immune checkpoint inhibitor therapy (Subgroups 1, 3, 5, and 7). (A) Subgroup 1. (B) Subgroup 3. (C) Subgroup 5. (D) Subgroup 7. For each subgroup, the curves compare patients who received short‐ (red line), intermediate‐ (blue line), or long‐ (green) courses immune checkpoint inhibitor therapy. For each panel, the four plots from left to right show the training dataset, external validation dataset 1, external validation dataset 2, and the pooled (combined) dataset.

In the external validation datasets, when applying the same subgroup definitions as in the training cohort, comparative analyses among patients recommended for long‐course ICI therapy showed that Subgroups 3 and 5 derived longer OS with long‐duration therapy relative to short or intermediate courses (*p* < 0.050; Table 3). In contrast, Subgroups 1 and 7 exhibited heterogeneous OS patterns across the three treatment durations, with no statistically significant difference in survival according to treatment duration (Figure 3 and Table 3). In the pooled analysis of all three cohorts, patients in Subgroup 3 who received long‐course therapy had markedly longer OS (*n* = 78; median 27.0 months, IQR 21.1–42.7 months) than those treated with an intermediate course (*n* = 69; median 17.6 months, IQR 11.0–26.9 months, *p* < 0.001) or short‐course (*n* = 89; median 7.9 months, IQR 5.3–16.8 months, *p* < 0.001, Figure 3). Similarly, the pooled analysis of patients in Subgroup 5 showed that patients treated with long‐course therapy achieved significantly better OS (*n* = 53; median 22.0 months, IQR 16.8–38.6 months) than those who received intermediate course (*n* = 58; median 11.4 months, IQR 6.8–23.7 months, *p* < 0.001) or short‐course (*n* = 90; median 9.3 months, IQR 3.6–20.6 months, *p* < 0.001, Figure 3). Notably, for each duration category, the sample sizes of Subgroups 3 and 5 exceeded the minimum requirements estimated by the power calculations.

For the remaining three subgroups (2, 4, and 6), the OPST framework identified survival patterns that were more frequently associated with short‐course therapy (one to three cycles). In the training cohort, Subgroups 4 and 6 achieved significantly longer OS with short‐course therapy than with intermediate‐ or long‐course therapy (all *p* < 0.05; Figure 4 and Table 4). However, in Subgroup 2, those who received short‐course therapy did not have significantly longer OS than those who received intermediate‐ or long‐course therapy, and OS was comparable across duration groups (Table 4).

**FIGURE 4:**
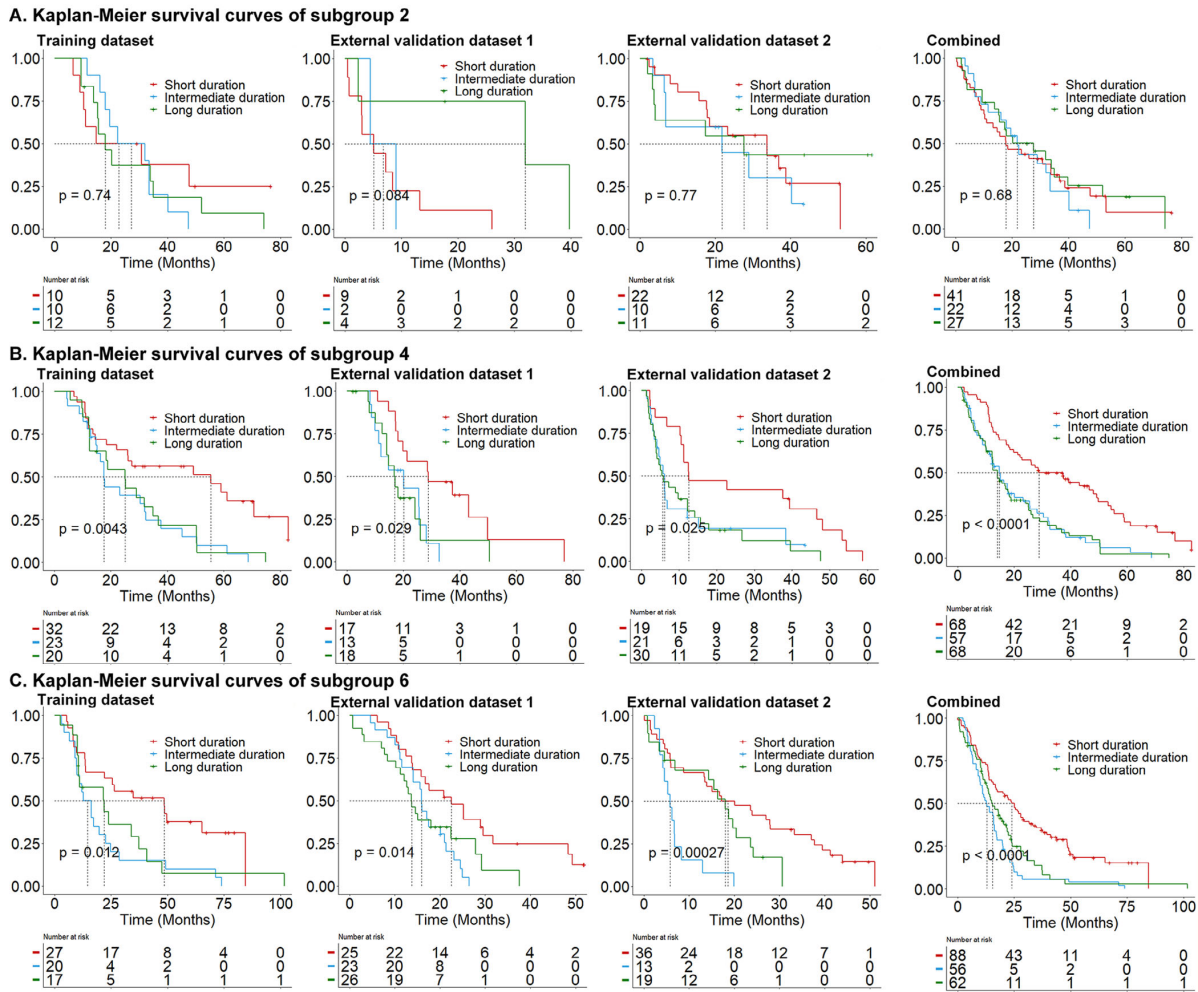
Kaplan–Meier overall survival curves for Optimal Policy Survival Tree (OPST)‐defined subgroups associated with short‐course immune checkpoint inhibitor therapy (Subgroups 2, 4, and 6). (A) Subgroup 2. (B) Subgroup 4. (C) Subgroup 6. For each subgroup, the curves compare patients who received short‐ (red line), intermediate‐ (blue line), or long‐ (green line) courses immune checkpoint inhibitor therapy. For each panel, the four plots from left to right show the training dataset, external validation dataset 1, external validation dataset 2, and the pooled (combined) dataset.

**TABLE 4:** Comparative analysis of overall survival in three OPST‐defined subgroups associated with short‐course immunotherapy. *p* value was calculated by log‐rank test with Benjamini‐Hochberg correction.

	Subgroup 2			Subgroup 4			Subgroup 6		
	*N*	Median (IQR)	*p*	*N*	Median (IQR)	*p*	*N*	Median (IQR)	*p*
**Training dataset**									
1–3 cycles	10	22.0 (10.6, 43.5)	Ref	32	30.1 (14.3, 59.5)	Ref	27	34.8 (13.5, 55.2)	Ref
4–6 cycles	10	27.2 (18.4, 33.5)	0.830	23	17.5 (12.2, 32.2)	0.011	20	14.5 (9.7, 23.2)	0.012
> 6 cycles	12	16.7 (12.9, 34.1)	0.830	20	22.0 (12.5, 35.4)	0.015	17	12.6 (10.5, 34.1)	0.044
**External validation dataset 1**									
1–3 cycles	9	5.1 (3.0, 8.5)	Ref	17	28.8 (18.1, 38.2)	Ref	25	21.0 (13.7, 29.5)	Ref
4–6 cycles	2	6.7 (5.6, 7.9)	0.940	13	18.0 (11.3, 25.4)	0.029	23	15.9 (11.3, 20.5)	0.016
> 6 cycles	4	24.8 (13.9, 33.8)	0.100	18	15.7 (10.7, 19.9)	0.040	26	13.8 (10.2, 20.1)	0.040
**External validation dataset 2**									
1–3 cycles	22	23.9 (11.5, 36.6)	Ref	19	12.6 (10.4, 42.9)	Ref	36	21.9 (6.7, 37.6)	Ref
4–6 cycles	10	20.5 (6.8, 27.1)	0.770	21	5.8 (3.5, 12.2)	0.040	13	5.8 (4.2, 6.8)	< 0.001
> 6 cycles	11	25.3 (3.9, 36.0)	0.770	30	5.6 (3.4, 13.7)	0.030	19	16.4 (4.5, 22.0)	0.044

Across the external validation cohorts, Subgroups 4 and 6 continued to demonstrate more favorable survival associations with short‐course therapy, yielding significantly longer OS than intermediate‐ or long‐course therapy (all *p* < 0.05; Table 4). For Subgroup 2, short‐course therapy achieved OS comparable to that of longer‐course therapy (*p* > 0.05; Table 4). Pooled analysis confirmed these findings. In Subgroup 4, short‐course therapy (*n* = 68) produced a median OS of 28.6 months (IQR 12.6–46.9 months), significantly outperforming intermediate‐course (*n* = 57, 12.8 months, IQR 6.2–23.7 months) and long‐course (*n* = 68, 12.5 months, IQR 5.6–22.3 months, all *p* < 0.001, Figure 4) therapy. Similarly, patients in Subgroup 6 receiving short‐course therapy (one to three cycles, *n* = 88) showed longer observed median OS values (24.5 months, IQR 12.6–41.6 months) than those receiving intermediate‐ (12.9 months, IQR 6.8–19.9 months, *n* = 56) or long‐course therapy (14.6 months, IQR 10.1–22.4 months, *n* = 62, all *p* < 0.001, Figure 4). All subgroup sample sizes exceeded the thresholds required, as determined by power calculations.

Analysis of potential confounders demonstrated that, in Subgroups 3 and 5 (long‐course therapy recommended) and Subgroups 4 and 6 (short‐course therapy recommended), distributions of clinical stage, ECOG score, and the number of concurrent chemotherapy/neoadjuvant cycles did not differ significantly among the three treatment duration groups (all *p* > 0.100; Tables S1–S3).

### 2.3 Sensitivity and Specificity Analysis

We conducted a series of sensitivity analyses to assess the utility and stability of the OPST framework for patient stratification across different clinical settings. With respect to over‐/undertreatment metrics, among patients without prolonged benefit, the OPST showed sensitivities of 55.3% (103/186), 61.1% (99/162), and 45.8% (82/179) in the training dataset and external validation datasets 1 and 2, respectively (the denominators were derived from dichotomizing each dataset according to the median OS cutoff). Among patients with prolonged benefit, the specificity values were 48.3% (90/186), 30.2% (49/162), and 25.1% (52/179) in the training dataset and external validation datasets 1 and 2, respectively. Overall, among patients assigned to long‐course therapy, 53.9% (284/527) did not achieve a prolonged OS benefit, indicating limited incremental value of extended treatment in this patient phenotype. Conversely, 36.2% (191/527) would have achieved prolonged benefit with short‐course therapy, suggesting that overtreatment could be avoided for these patients.

Landmark analyses showed stage‐dependent associations between treatment duration and survival. At the three‐cycle landmark, continuation beyond short‐course therapy was not associated with improved OS across all cohorts. In contrast, at the six‐cycle landmark, continuation to long‐course therapy was associated with significantly improved survival compared with discontinuation after intermediate‐course therapy (all *p* < 0.001; Figure S10). Within the subset of patients with available toxicity and treatment discontinuation records, the distributions of immune‐related adverse events (irAEs) grades and treatment discontinuation reasons were broadly comparable across short‐, intermediate‐, and long‐course treatment groups, with no consistent differences observed across cohorts (Tables S6–S11). Survival analyses stratified by irAE grades and discontinuation categories showed heterogeneous and non‐reproducible patterns across datasets (Figure S11). Survival patterns across both adverse events (Figure S11A–C) and discontinuation categories (Figure S11D–F) were heterogeneous.

A post hoc analysis evaluating a potential safe discontinuation time showed progressive convergence in OS between patients treated for one to three cycles and those treated for more than six cycles. The absolute difference in OS rates declined from 16.5% at 1 year (55.6% vs. 72.1%) to 3.0% at 3 years (18.9% vs. 21.9%) and 1.2% at 5 years (5.6% vs. 6.8%) for short‐ and long‐course therapy, respectively.

### 2.4 Extended and Endpoint‐Specific OPSTs

Extended OPST models by incorporating additional variables and endpoints were developed to evaluate the stability of the proposed treatment duration stratification framework. For the extended OPST using six features, the tree topology remained structurally identical to the original, with only modest upward shifts in the first‐level dNLR split (from 2.100 to 2.395) and the terminal age split (from 64.5 to 66.5 years; Figure S1). Consistent with the primary analysis, patients in Subgroups 3 and 5 benefitted the most from long‐course therapy across all cohorts (Figure S2; *p* < 0.001), whereas patients in Subgroups 4 and 6 benefitted the most from short‐course therapy (Figure S2; *p* < 0.001).

OPSTs optimized separately for the 3‐year and 5‐year OS endpoints (constructed using the original three variables) reproduced the structure of the original tree (Figures S3 and S5). In the 3‐year OPST, patients in Subgroups 2, 4, and 6 had a 3‐year OS rate of 29.9% (44/147) with short‐course therapy, which exceeded that of intermediate‐course (11.4%, 12/105; *p* = 0.007) and long‐course (11.2%, 14/124; *p* = 0.003) therapy. In the 5‐year OPST, patients in these subgroups who received short‐course therapy had a better 5‐year OS rate (7.2%, 19/263) than those who received intermediate‐course (1.5%, 3/194; *p* = 0.013) and long‐course (3.1%, 6/192; *p* = 0.111) therapy. Conversely, in Subgroups 1, 3, 5, and 7, those patients who received long‐course therapy had the highest 3‐year survival rate (long‐course: 28.6%, 57/199; intermediate‐course: 8.3%, 16/193; and short‐course: 13.3%, 38/286; long‐ vs. intermediate‐course *p* = 0.039; and long‐ vs. short‐course *p* < 0.001) and at 5‐year OS rate (long‐course: 12.2%, 16/131; intermediate‐course: 2.9%, 3/104; and short‐course 2.9%, 5/170; long‐ vs. intermediate‐course *p* = 0.029; and long‐ vs. short‐course *p*  =  0.007).

In the subset of patients with available PD‐L1 expression data, incorporation of PD‐L1 expression into model construction produced a tree structure highly consistent with the original OPST model, and the seven‐subgroup architecture was preserved (Figure S7). The primary modification was limited to the terminal level of selected branches, where PD‐L1 expression replaced one of the original splitting variables in certain leaf nodes. Kaplan–Meier survival curves for the altered key subgroups are presented in Figure S8.

### 2.5 Comparison With External Controls

We compared survival outcomes between OPST‐recommended short‐course and long‐course patient groups across different treatment regimens to evaluate the applicability of OPST‐guided duration strategies in varied therapeutic contexts. Patients adhering to the OPST‐recommended short course achieved significantly longer OS than either the ICI or non‐ICI control cohorts (*p* < 0.001; Figure S9A). Those exceeding the recommended duration experienced inferior OS to compliant patients (*p* < 0.001). Although initially outperforming the ICI control for < 50 months, their survival curve eventually converged with the control, a crossover that was not observed among compliant patients receiving short‐course therapy. The median OS was 25.2 months in OPST‐compliant patients versus 14.6 months for those receiving a longer course (hazard ratio [HR]: 0.56, 95% CI: 0.46–0.69, *p* < 0.001).

Similarly, patients who completed the OPST‐recommended long‐course therapy achieved superior OS to both control cohorts (*p* < 0.001; Figure S9B). Truncating therapy below the recommended duration eliminated this advantage, with a median OS of 11.2 versus 25.1 months (HR: 0.46, 95% CI: 0.38–0.56, *p* < 0.001).

## 3 Discussion

In this retrospective multicenter study, we built an interpretable AI framework that relies on three readily obtainable baseline variables, age, maximum tumor diameter, and dNLR, to personalize decisions regarding the duration of ICI therapy in patients with NSCLC. Validation in two external cohorts from China and the United States confirmed the approach. The OPST identified seven biological subgroups (phenotypes), four of which had clear, duration‐specific survival advantages. In particular, patients in Subgroups 4 and 6 (37.8%) derived their greatest OS benefit from short‐course therapy (one to three cycles), whereas Subgroups 3 and 5 (41.5%) benefitted most from long‐course therapy (more than six cycles). Guided by the Hippocratic maxim “first, do no harm,” we quantified overtreatment‐associated patterns within the OPST framework and observed that a subset of patients (18.1%) assigned to short‐course therapy maintained prolonged survival outcomes, suggesting the potential feasibility of reducing unnecessary treatment exposure in selected populations.

Defining an evidence‐based stopping point for ICIs is critical both to mitigate irAEs and to reduce the substantial financial burden on patients. Although clinical trials usually recommend a maximum of 35 cycles and report improved tumor‐control rates with extended therapy, they have not conclusively demonstrated an OS advantage [8, 10, 12, 13, 15]. Leveraging real‐world data from different countries, our three‐factor, fully interpretable model consistently stratified patients across geographically distinct datasets and retained its structure when expanded to include invasive pathological subtype, clinical stage, and smoking status [8, 9, 14, 15] as covariates. Notably, retraining the OPST to optimize 3‐year or 5‐year OS, or incorporating PD‐L1 expression into the model, did not alter the resulting decision tree, underscoring the robustness and generalizability of our approach.

The median of five ICI cycles observed in our study is lower than that reported in pivotal trials [8, 9, 15] but aligns with contemporary real‐world study series from major cancer centers, where early discontinuation is common because of progression, toxicity, or economic constraints [8, 18, 19]. A recent study on post‐discontinuation survival from the Dana‐Farber Cancer Institute reported a median number of ICI therapy cycles ranging between 2.3 and 7.2 [10]. These data corroborate our finding that short‐course treatment is frequently practiced and can still yield favorable outcomes in patients with NSCLC.

Although current clinical guidelines have not yet correlated short‐course ICI therapy with durable OS benefit of NSCLC, the modest predictive power of current biomarkers, the cumulative risk of immune‐related toxicities, and persistent uncertainty about long‐term outcomes all point to a need to reconsider the therapeutic paradigm. Our study provides real‐world evidence supporting such a shift. The subgroup division criteria of OPST are consistent with the age, LD, and dNLR cutoff values mentioned in previous studies [8, 9, 14, 15, 16]. Using a unified OPST applied to patients from six Chinese centers and the MSKCC in New York, we reproducibly delineated seven clinicopathological subgroups whose definitions were preserved across the original, extended, and 3‐ and 5‐year OS versions of the OPST. Subgroups 3, 4, 5, and 6, comprising 79.3% of all the patients, derived a statistically significant OS advantage from the OPST‐recommended treatment duration in both the training and external validation sets. Subgroups 1 and 7, for which the OPST advised long‐course therapy, did not show uniform significance across individual datasets; however, in the pooled analysis, long‐course therapy still yielded a superior OS compared with shorter course regimens. Our findings suggest that NSCLC patients who simultaneously exhibit small tumors with low dNLR values and those who simultaneously harbor large tumors with high dNLR values—despite representing opposite ends of the immunotherapy prognostic spectrum—long‐course ICI therapy is associated with better OS [20]. In the combined analysis, Subgroup 2, characterized by dNLR < 2.1 and LD 9.5–26.5 mm, represents a phenotype in which treatment duration appears to have limited influence on survival. In this subgroup, short‐, intermediate‐, and long‐course immunotherapy yielded comparable outcomes, although the analysis may be underpowered to detect modest differences. These findings suggest that treatment duration may be less critical in this subgroup, and that shorter treatment courses could be considered in selected patients, while acknowledging the need for further validation. This pattern also indicates that the impact of treatment duration is not uniform across patient phenotypes. Collectively, these findings support further investigation of phenotype‐specific immunotherapy duration patterns and suggest that the OPST framework may serve as a decision‐supportive reference for future clinical evaluation across diverse patient profiles.

This study has some limitations. First, because the vast majority of included patients had an ECOG performance status of 1 and received first‐ or second‐line therapy, these variables showed limited variability and were unlikely to provide stable or informative contributions to model construction. Future prospective datasets with more balanced distributions of these factors are required for more definitive evaluation. Second, several supplementary analyses were performed in selected subsets rather than the full study population because of incomplete real‐world data capture across centers. In particular, irAE records, treatment discontinuation reasons, and PD‐L1 testing results were not uniformly available for all patients owing to heterogeneity in clinical documentation and testing practices. These factors may introduce additional selection bias and limit the generalizability of subset‐based analyses. Although the PD‐L1 sensitivity analysis demonstrated structural consistency with the original framework, the OPST approach should be considered complementary to the established biomarker‐guided immunotherapy decision‐making. The sensitivity analysis results on immune‐related toxicity and discontinuation patterns were broadly comparable across treatment groups and showed inconsistent associations with survival, indicating they are unlikely to fully explain the observed findings. However, given the observational design, residual confounding cannot be excluded; the current findings should not be interpreted as sufficient to justify routine early discontinuation or extension of ICI therapy in clinical practice. Given the heterogeneity of real‐world treatment settings, including retreatment, response after rechallenge, and rebound progression, prospective validation remains necessary before clinical implementation of the OPST framework. Third, although post‐treatment response categories (complete, partial, stable, or progressive disease) are known to influence survival [21, 22, 23], our goal was to create a pre‐treatment tool. Future research should consider variables measured during therapy. Finally, despite major confounders, including clinical stage, ECOG score, and combination‐therapy cycles [24, 25], validation showed no significant difference across the subgroups (*p* > 0.120); unmeasured factors, including treatment response dynamics, physician decision‐making, socioeconomic considerations, and other aspects of real‐world clinical care, may still influence both treatment duration and survival outcomes. Broader validation is warranted to validate the utility of our recommendations for guiding the duration of ICI therapy in these subgroups. A prospective observational study (Research Registry: 11873) has been initiated to evaluate the real‐world performance of the interpretable framework. The study includes predefined efficacy and long‐term endpoints, including OS and PFS, to assess both the clinical benefit and safety of OPST‐guided duration strategies. Through the incorporation of prospectively collected data, future validation efforts may help address the inherent limitations of retrospective observational analyses and further evaluate the potential clinical utility of the OPST framework.

Our interpretable OPST framework provides a clinically interpretable strategy for evaluating phenotype‐associated treatment duration patterns in NSCLC, and identifies a subset of patients potentially associated with limited incremental survival benefit from prolonged treatment exposure. Further prospective studies are warranted to determine whether this framework can be integrated into clinically applicable precision immunotherapy strategies.

## 4 Materials and Methods

### 4.1 Study Design and Participants

This retrospective, multicenter observational study enrolled consecutive patients with pathologically confirmed NSCLC who initiated ICI therapy, either as monotherapy or in combination regimens, between May 1, 2016, and December 31, 2024, at six hospitals in China and the MSKCC in New York. The eligibility criteria were as follows: (i) ≥ 1 cycle of ICI therapy and (ii) documented OS follow‑up. Patients were excluded if they (i) had incomplete baseline data, (ii) non‑primary lung malignancies, or (iii) missing information on treatment duration. All therapies adhered to current clinical guidelines. The decision to continue or discontinue ICIs was made jointly by the physicians and patients. The study complied with the Declaration of Helsinki, received institutional review board approval at each site, and, owing to its retrospective nature, was granted a waiver of written informed consent. The results were reported according to the STROBE guidelines.

### 4.2 Data Collection

Baseline clinical variables with potential prognostic relevance were abstracted from electronic health records for inclusion as candidate modeling factors. In this study, data on PD‐L1 expression and TMB were not available for all enrolled patients, because its assessment requires invasive pathological punctures, which would be contrary to the objective of this study of establishing a clinically simple method using routinely available data.

The primary endpoint was OS, defined from ICI therapy initiation to death from any cause; patients alive on the final follow‐up date (March 31, 2025) were censored. We did not use PFS as the endpoint in this study because, in clinical practice, extended ICI therapy duration may be associated with longer PFS, whereas the relationship between ICI therapy duration and OS remains complex and warrants further investigation [8, 26, 27].

In addition to the seven participating centers, two external control cohorts were incorporated. An ICI control comprised published datasets of patients with NSCLC treated with ICI therapy [28, 29, 30, 31], and a non‐ICI control consisted of patients with NSCLC from MSKCC who did not receive ICIs [31]. Neither control cohort reported treatment duration, and the control cohorts were used to compare OS between (i) patients who followed the recommendation that the model proposed in this study and (ii) patients who did not.

### 4.3 Counterfactual Random Forest‐Based Reward‐Estimation Matrix

In routine clinical practice, when a patient is given a specific treatment regimen, a clear survival outcome can be observed for the patient. However, to estimate potential survival outcomes under different ICI treatment durations requires observing outcomes for each potential treatment duration in the same patient. However, this is not feasible in real‐world clinical settings, and thus, counterfactual outcomes need to be inferred, that is, we estimated the potential OS if a given patient had been treated with a different number of ICI therapy cycles.

To estimate the survival benefit that an individual patient might have achieved under alternative treatment durations, we trained a counterfactual random forest model [32]. The model used three easily measured baseline variables, age, maximum tumor diameter (LD), and dNLR [33, 34, 35, 36], which have recognized prognostic value in ICI therapy, together with the actual number of ICI cycles. LD was recorded in millimeters from cross‐sectional imaging, and dNLR was calculated from routine blood counts. The model generated a reward‐estimation matrix giving the predicted OS for each potential treatment duration in each patient. To promote parsimony and clinical usability [37], no additional covariates were included for the original reward‐estimation matrix building. Details of the construction of the counterfactual random forest and reward matrix are presented in Supporting Information.

According to the enrolled patients in the study, 84.0% (885/1054) received ICIs with chemotherapy or other recommended regimens and only one patient exceeded 32 cycles. Modeling each cycle count as a potential treatment duration option would have been impractical due to data sparsity. Therefore, we grouped treatment length into three pragmatic categories of treatment duration, reflecting clinical trial designs and real‐world practice [10, 25, 38, 39, 40]: short‐ (one to three cycles), intermediate‐ (four to six cycles), and long‐course (more than six cycles).

### 4.4 OPST

Using the reward‐estimation matrix from the training dataset, we built an OPST using Interpretable AI, version 3.2.1 (https://www.interpretable.ai/). Each terminal node of the OPST defined a patient subgroup with homogeneous baseline characteristics and an associated ICI treatment‐duration pattern (short, intermediate, or long). Hyperparameters for the random forest and OPST, number of trees, maximum depth, and minimum node size were tuned by fivefold cross‐validation.

After finalizing the OPST on the training dataset, the same patient grouping strategy was maintained and applied to two independent validation datasets. In accordance with recommendations from the Interpretable AI framework, the sample size of each validation dataset was required to be similar to or larger than that of the training dataset. Therefore, patients from the Guangdong Provincial People's Hospital formed the training dataset. External validation dataset 1 comprised patients from The First Affiliated Hospital of China Medical University, Shengjing Hospital of China Medical University, The First Affiliated Hospital of Zhengzhou University, and The First Affiliated Hospital of University of Science and Technology of China. External validation dataset 2 comprised patients from Nanfang Hospital of Southern Medical University and MSKCC in New York.

### 4.5 Sensitivity and Specificity Analysis

To evaluate potential patterns of prolonged or limited treatment exposure identified by the OPST framework, we used the median OS of 15.8 months as the cut‐off, derived from previous related studies and the actual OS outcomes of patients in our study [41, 42]. Patients with OS greater than 15.8 months were classified as having improved OS, whereas those with OS below the cutoff were classified as not having improved OS. According to a definition used in a previous study [43], undertreatment (i.e., sensitivity) was defined as the proportion of patients with non‐improved OS for whom long‐course ICI therapy was recommended by the OPST, divided by the sum of both these patients and those with non‐improved OS for whom long‐course treatment had not been recommended by the OPST. Conversely, overtreatment (i.e., specificity) was defined as the proportion of patients with improved OS for whom long‐course ICI therapy was not recommended by the OPST, divided by the sum of these patients and those with improved OS for whom the OPST had recommended long‐course ICI therapy.

To further assess potential bias arising from non‐random treatment duration and unmeasured confounding in real‐world settings, we performed additional analyses. First, landmark analyses were conducted at three and six cycles of ICI therapy to mitigate time‐dependent bias by restricting analyses to patients alive at each landmark time point. Second, irAEs and treatment discontinuation reasons were systematically collected and harmonized across cohorts. irAEs were categorized by grade (G1–G4), and discontinuation reasons were classified into four predefined groups: irAEs, disease progression, treatment completion or conversion, and others (details see Supporting Information). Third, within each OPST‐defined subgroup, we compared the distributions of irAE grades and discontinuation reasons across treatment duration categories.

### 4.6 Statistical Analysis

Continuous variables are summarized as medians with IQR; categorical variables as counts (percentages). Random forest and OPST models were implemented in Julia (version 1.10.5, https://julialang.org/) and R (version 4.3.1, R Foundation for Statistical Computing, Vienna, Austria). Group differences in OS were assessed using Kaplan–Meier survival analysis and log‐rank tests with HR. An extended OPST incorporating six variables (age, smoking status, pathological subtype, clinical stage, LD, and dNLR) was trained on counterfactual predictions and validated analogously. To evaluate treatment‐duration patterns associated with higher survival rates, separate OPSTs were also constructed and optimized for 3‐year and 5‐year OS endpoints at the 3‐ and 5‐year OS endpoints. In addition, an extended OPST model incorporating PD‑L1 expression was developed and validated using patients with complete data to verify the stability of the method proposed in this study. A post hoc analysis compared OS between short‐ and long‐course ICI therapy groups to explore a potential treatment discontinuation time point associated with converging survival outcomes. The sample size calculations for OPST‐defined subgroups were estimated using PASS (version 15.0). All statistical tests were two‐sided with *p* < 0.05 regarded as significant.

## Author Contributions

Guarantor of integrity of the entire study: J. S., Jianhua L., and X. Q. Study concepts and design: S. W., N. X., Q. N., and Q. H. Literature research: S. W., Jianhua L., Q. N., R. S., A. A., N. X., Q. H., G. Y., Jingyi L., Q. H., K. D., and L. W. Clinical studies: G. Y., Jianhua L., Q. H., K. D., L. W., T. A., and X. Q. Experimental studies/data analysis: S. W., Jianhua L., Q. N., R. S., and A. A. Statistical analysis: R. S., N. X., Q. H., G. Y., and J. S. Manuscript preparation: S. W., Jianhua L., Q. N., R. S., A. A., N. X., Q. H., G. Y., Jingyi L., Q. H., K. D., L. W., T. A., X. Q., and J. S. Manuscript editing: S. W., Jianhua L., Q. N., R. S., A. A., N. X., Q. H., G. Y., Jingyi L., Q. H., K. D., L. W., T. A., X. Q., and J. S. All authors have read and approved the final manuscript.

## Funding

This work was supported by the National Natural Science Foundation of China (92259104), Scientific Research Fund of Liaoning Provincial Education Department (LJ242410159058), and the Xingliao Talent Program of Liaoning Province (XLYC2403102). The funders had no role in the study design, data collection and analysis, decision to publish, or preparation of the manuscript. The corresponding author confirms that he had full access to all the study data and had the final responsibility for deciding to submit the manuscript for publication.

## Ethics Statement

This study was approved by the ethics committee of The First Affiliated Hospital of China Medical University, The First Affiliated Hospital of University of Science and Technology of China, Guangdong Provincial People's Hospital, Zhuhai People's Hospital, and Nanfang Hospital of Southern Medical University (KY2025‐473‐02, AF‐SOP‐07‐1, 2023‐RE‐113, NFEC‐2024‐423, and 2025‐KT‐145). The requirement for written informed consent was waived owing to the use of de‐identified and retrospectively collected data.

## Conflicts of Interest

The authors declare no conflicts of interest.

## Supporting information




**Supporting file 1**: mco271032‐sup‐0001‐SuppMat.docx

## Data Availability

The data that support the findings of this study are available from the corresponding authors with a signed data access agreement. The raw image and follow‐up data are not publicly available because they contain sensitive information that could compromise patient privacy.
