# Supplementary material for: Optimal, Interpretable, and Personalized Immune Checkpoint Inhibitor Duration in Non‐Small Cell Lung Cancer: A Multicenter Real‐World Study
Source: MedComm (2020). 2026 Sep 29;7(10):e71032. doi: 10.1002/mco2.71032 (PMC13620853; doi:10.1002/mco2.71032)
Supplement: Supplementary file 1 [file MCO2-7-e71032-s001.docx]

**Optimal, interpretable, and personalized immune checkpoint inhibitor duration in non-small cell lung cancer: a multicenter real-world study**

**Authors and Affiliations**

Siqi Wang^1^^#^ MD, Jianhua Liu^2#^ MD, Qiang Nie^3#^ MD, Ruichuan Shi^4#^ MD, Nan Xu^1^ MD, Qianqian He^1^ MD, Guanchao Ye^5^ MD, Jingyi Liu^6^ MD, Qiuyang Hou^7^ MD, Kexue Deng^7^ MD, Lu Wang^8^* MD, Taixue An^9^* MD, Anyi An^10^* MD, Xiujuan Qu^4^* MD, and Jiangdian Song^1^* PhD.

^#^ The authors contributed equally to this study.

*Co-corresponding authors

1. School of Health Management, China Medical University, Shenyang, Liaoning 110122 China
2. Department of Oncology, Guangdong Provincial People’s Hospital (Guangdong Academy of Medical Sciences), Southern Medical University, Guangzhou 510080, China
3. Department of Pulmonary Surgery, Guangdong Lung Cancer Institute, Guangdong Provincial People’s Hospital (Guangdong Academy of Medical Sciences), Southern Medical University, Guangzhou 510080, China
4. Department of Medical Oncology, the First Hospital of China Medical University, Shenyang, Liaoning Province, China
5. Department of Thoracic Surgery, the First Affiliated Hospital of Zhengzhou University, Zhengzhou, Henan, China
6. The First Hospital of China Medical University, Shenyang, Liaoning,110001, China
7. Department of Radiology, The First Affiliated Hospital of University of Science and Technology of China (USTC), Division of Life Sciences and Medicine, USTC, Hefei, Anhui, 230036, China
8. Shengjing hospital of China Medical University, Shenyang, Liaoning, 110022, China
9. Department of Laboratory Medicine, Nanfang Hospital, Southern Medical University, Guangzhou, 510515, China
10. Jinzhou Medical University, Jinzhou, Liaoning Province, China

**Corresponding Author:**

Prof. Jiangdian Song

China Medical University

No.77 Puhe Road, Shenbei New District, Shenyang, Liaoning, 110122 China

Email: [jdsong@cmu.edu.cn](mailto:jdsong@cmu.edu.cn), song.jd0910@gmail.com

**Figure S1. Extended Optimal Policy Survival Tree (six-feature model) for tailoring immunotherapy duration in NSCLC.** The tree partitions patients into seven terminal leaves on the basis of derived neutrophil-to-lymphocyte ratio (dNLR), longest tumor diameter (LD), and age. **Subgroup 1:** dNLR < 2.395 and LD < 9.5 mm. **Subgroup 2:** dNLR < 2.395 with intermediate tumor size (9.5 ≤ LD < 26.5 mm). **Subgroup 3:** dNLR < 2.395 and large tumors (LD ≥ 26.5 mm). **Subgroup 4:** dNLR ≥ 2.395, LD < 48.5 mm, and age < 66.5 years. **Subgroup 5:** dNLR ≥ 2.395, LD < 48.5 mm, and age ≥ 66.5 years. **Subgroup 6:** dNLR 2.395–4.97 with bulky disease (LD ≥ 48.5 mm). **Subgroup 7:** dNLR ≥ 4.97 and LD ≥ 48.5 mm.

**Figure S2. Kaplan–Meier survival curves for the six-feature, extended Optimal Policy Survival Tree.** Survival outcomes for NSCLC patients are shown according to immunotherapy course length as determined by the extended OPST built on six baseline variables. As in the original model, patients in subgroups 3 and 5 achieve their best overall survival with long-duration therapy (>6 cycles), whereas patients in subgroups 4 and 6 attain optimal benefit with a short-duration course (≤3 cycles).

**Figure S3. Three-year overall survival Optimal Policy Survival Tree for immunotherapy duration in NSCLC.** The subgroup splits and tree architecture are consistent with those of the original OPST and the six-feature model shown in Figure S1; therefore, branch details are not repeated here.

**Figure S4. Kaplan–Meier survival curves derived from the 3-year overall survival Optimal Policy Survival Tree.** Survival is plotted for patients with NSCLC according to the treatment-duration patterns identified by the 3-year OPST. Consistent with the original tree, patients in Subgroups 3 and 5 showed more favorable overall survival associations with long-duration immunotherapy (>6 cycles), whereas patients in Subgroup 6 showed more favorable survival associations with short-duration therapy (≤3 cycles). In Subgroup 4, short-duration therapy was not associated with a statistically significant survival advantage over longer regimens, and differences among the three duration groups were not significant (P = 0.23).

**Figure S5. Five-year overall survival Optimal Policy Survival Tree for immunotherapy duration in NSCLC.** The subgroup splits and tree architecture are consistent with those of the original OPST and the six-feature model shown in Figure S1; therefore, branch details are not repeated here.

**Figure S6. Kaplan–Meier survival curves generated from the 5-year overall survival Optimal Policy Survival Tree.** For this 5-year endpoint, the tree again identified Subgroup 3 as showing more favorable survival associations with long-duration immunotherapy (>6 cycles), whereas Subgroup 4 showed more favorable outcomes with short-duration therapy (≤3 cycles). In Subgroups 5 and 6, the OPST-identified duration patterns were not associated with a statistically significant survival advantage over alternative regimens (log-rank P = 0.48 and P = 0.34, respectively), likely because the smaller number of patients surviving to five years reduced statistical power for subgroup comparisons.

**Figure S7. Optimal Policy Survival Tree incorporating PD-L1 expression for overall survival stratification.** Subgroup 1 included patients with lower dNLR (<2.1), PD-L1 negative expression (<1%), and smaller longest diameter (LD < 9.5 mm). Subgroup 2 included patients with lower dNLR (<2.1), PD-L1 negative expression (<1%), and larger longest diameter (LD ≥ 9.5 mm). Subgroup 3 included patients with lower dNLR (<2.1) and PD-L1 positive expression (≥1%). Subgroup 4 included patients with higher dNLR (≥2.1), LD <48.5 mm, and age <64.5 years. Subgroup 5 included patients with higher dNLR (≥2.1), LD <48.5 mm, and age ≥64.5 years. Subgroup 6 included patients with dNLR ≥2.1, LD ≥48.5 mm, and PD-L1 negative expression (<1%). Subgroup 7 included patients with dNLR ≥2.1, LD ≥48.5 mm, and PD-L1 positive expression (≥1%).

**Figure S8. Kaplan–Meier overall survival curves for the OPST incorporating PD-L1 expression in key subgroups.** Only the key subgroups modified by the inclusion of PD-L1 (Subgroup 3 [A] and Subgroup 6 [B]) are shown for clarity. For each subgroup, survival curves compare patients receiving short-course (red), intermediate-course (blue), and long-course (green) ICI therapy. Each panel includes results from the training dataset and the pooled dataset.

**Figure S9. Kaplan–Meier overall survival curves benchmarked against ICI and non-ICI control cohorts**. (A) Survival in patients receiving OPST-identified short-course therapy (≤3 cycles) versus those receiving longer treatment, shown alongside the external ICI and non-ICI control cohorts. (B) Survival in patients receiving OPST-identified long-course therapy (>6 cycles) versus those receiving fewer cycles, compared with the external ICI and non-ICI control cohorts.

**Table S1.** **Assessment of clinical confounders across patients treated with different ICI courses in Subgroups 3, 4, 5, and 6 (training dataset).** Comparative analysis of baseline factors among patients received short-, intermediate-, and long-course immunotherapy; P values indicate statistical differences across the three categories. Note that some variables were missing in either the local cohort or the MSK dataset, and certain subgroups are not shown.

|  | Short-course | Intermediate- course | Long- course | P |
| --- | --- | --- | --- | --- |
|  | Number | Number | Number |  |
| **Subgroups 3** |  |  |  |  |
| Clinical stage |  |  |  | 0.171 |
| IA-IIIC | 49 | 26 | 33 |  |
| IV | 5 | 8 | 5 |  |
| ECOG score |  |  |  | 0.829 |
| 0-1 | 40 | 25 | 30 |  |
| 2 | 14 | 9 | 8 |  |
| Combined therapy |  |  |  | 0.993 |
| 1-2 | 35 | 22 | 25 |  |
| >2 | 19 | 12 | 13 |  |
| **Subgroups 4** |  |  |  |  |
| Clinical stage |  |  |  | 0.133 |
| IA-IIIC | 31 | 19 | 19 |  |
| IV | 1 | 4 | 1 |  |
| ECOG score |  |  |  | 0.258 |
| 0-1 | 27 | 15 | 15 |  |
| 2 | 5 | 8 | 5 |  |
| Combined therapy |  |  |  | 0.632 |
| 1-2 | 25 | 18 | 26 |  |
| >2 | 7 | 5 | 4 |  |
| **Subgroups 5** |  |  |  |  |
| Clinical stage |  |  |  | 0.478 |
| IA-IIIC | 19 | 10 | 10 |  |
| IV | 1 | 2 | 2 |  |
| ECOG score |  |  |  | 0.767 |
| 0-1 | 17 | 10 | 9 |  |
| 2 | 3 | 2 | 3 |  |
| Combined therapy |  |  |  | 0.593 |
| 1-2 | 12 | 5 | 6 |  |
| >2 | 8 | 7 | 6 |  |
| **Subgroups 6** |  |  |  |  |
| Clinical stage |  |  |  | 0.683 |
| I-IIIC | 24 | 16 | 14 |  |
| IV | 3 | 4 | 3 |  |
| ECOG score |  |  |  | 0.803 |
| 0-1 | 20 | 15 | 12 |  |
| 2 | 7 | 5 | 5 |  |
| Combined therapy |  |  |  | 0.217 |
| 1-2 | 19 | 10 | 8 |  |
| >2 | 8 | 10 | 9 |  |

**Table S2. Assessment of clinical confounders across patients treated with different ICI courses in Subgroups 3, 4, 5, and 6 (external validation dataset 1).** Comparative analysis of baseline factors among patients received short-, intermediate-, and long-course immunotherapy; P values indicate statistical differences across the three categories. Note that some variables were missing in either the local cohort or the MSK dataset, and certain subgroups are not shown.

|  | Short-course | Intermediate-course | Long-course | P |
| --- | --- | --- | --- | --- |
|  | Number | Number | Number |  |
| **Subgroups 3** |  |  |  |  |
| Clinical stage |  |  |  | 0.704 |
| IA-IIIC | 6 | 12 | 8 |  |
| IV | 6 | 8 | 4 |  |
| ECOG score |  |  |  | 0.858 |
| 0-1 | 7 | 10 | 7 |  |
| 2 | 5 | 10 | 5 |  |
| Combined therapy |  |  |  | 0.765 |
| 1-2 cycles | 5 | 11 | 6 |  |
| >2 cycles | 7 | 9 | 6 |  |
| **Subgroups 4** |  |  |  |  |
| Clinical stage |  |  |  | 0.211 |
| IA-IIIC | 11 | 12 | 13 |  |
| IV | 6 | 1 | 5 |  |
| ECOG score |  |  |  | 0.986 |
| 0-1 | 10 | 8 | 11 |  |
| 2 | 7 | 5 | 7 |  |
| Combined therapy |  |  |  | 0.494 |
| 1-2 cycles | 9 | 6 | 12 |  |
| >2 cycles | 8 | 7 | 6 |  |
| **Subgroups 5** |  |  |  |  |
| Clinical stage |  |  |  | 0.988 |
| IA-IIIC | 23 | 19 | 13 |  |
| IV | 13 | 11 | 8 |  |
| ECOG score |  |  |  | 0.120 |
| 0-1 | 24 | 25 | 12 |  |
| 2 | 12 | 5 | 9 |  |
| Combined therapy |  |  |  | 0.208 |
| 1-2 cycles | 25 | 16 | 10 |  |
| >2 cycles | 11 | 14 | 11 |  |
| **Subgroups 6** |  |  |  |  |
| Clinical stage |  |  |  | 0.725 |
| I-IIIC | 18 | 18 | 16 |  |
| IV | 7 | 6 | 9 |  |
| ECOG score |  |  |  | 0.320 |
| 0-1 | 15 | 10 | 12 |  |
| 2 | 11 | 13 | 13 |  |
| Combined therapy |  |  |  | 0.362 |
| 1-2 cycles | 11 | 12 | 15 |  |
| >2 cycles | 15 | 11 | 10 |  |

**Table S3.** **Assessment of clinical confounders across patients treated with different ICI courses in Subgroups 3, 4, 5, and 6 (external validation dataset 2).** Comparative analysis of baseline factors among patients received short-, intermediate-, and long-course immunotherapy; P values indicate statistical differences across the three categories. Note that some variables were missing in either the local cohort or the MSK dataset, and certain subgroups are not shown.

|  | Short-course | Intermediate-course | Long-course | P |
| --- | --- | --- | --- | --- |
|  | Number | Number | Number |  |
| **Subgroups 3** |  |  |  |  |
| Clinical stage |  |  |  | 0.962 |
| IA-IIIC | 3 | 9 | 15 |  |
| IV | 1 | 3 | 4 |  |
| ECOG score |  |  |  | 0.720 |
| 0-1 | 18 | 10 | 20 |  |
| 2 | 5 | 5 | 8 |  |
| Combined therapy |  |  |  | 0.818 |
| 1-2 cycles | 16 | 11 | 18 |  |
| >2 cycles | 7 | 4 | 10 |  |
| **Subgroups 4** |  |  |  |  |
| Clinical stage |  |  |  | 0.532 |
| IA-IIIC | 9 | 7 | 22 |  |
| IV | 4 | 5 | 7 |  |
| ECOG score |  |  |  | 0.369 |
| 0-1 | 10 | 15 | 21 |  |
| 2 | 9 | 6 | 9 |  |
| Combined therapy |  |  |  | 0.196 |
| 1-2 cycles | 11 | 13 | 24 |  |
| >2 cycles | 8 | 8 | 6 |  |
| **Subgroups 5** |  |  |  |  |
| Clinical stage |  |  |  | 0.232 |
| IA-IIIC | 3 | 9 | 8 |  |
| IV | 4 | 2 | 4 |  |
| ECOG score |  |  |  | 0.946 |
| 0-1 | 22 | 12 | 12 |  |
| 2 | 11 | 6 | 7 |  |
| Combined therapy |  |  |  | 0.718 |
| 1-2 cycles | 21 | 11 | 10 |  |
| >2 cycles | 12 | 7 | 9 |  |
| **Subgroups 6** |  |  |  |  |
| Clinical stage |  |  |  | 0.246 |
| I-IIIC | 8 | 6 | 6 |  |
| IV | 6 | 1 | 1 |  |
| ECOG score |  |  |  | 0.403 |
| 0-1 | 20 | 10 | 11 |  |
| 2 | 14 | 5 | 8 |  |
| Combined therapy |  |  |  | 0.566 |
| 1-2 cycles | 18 | 11 | 10 |  |
| >2 cycles | 16 | 4 | 9 |  |

**Table S4. Comparative analysis of overall survival across the four subgroups of patients recommended long‑course immunotherapy by OPST.** Both unadjusted p‑values (P-1) and p‑values adjusted using the Benjamini‑Hochberg method (P-2) are presented.

|  | Subgroup 1 | | | Subgroup 3 | | | Subgroup 5 | | | Subgroup 7 | | |
| --- | --- | --- | --- | --- | --- | --- | --- | --- | --- | --- | --- | --- |
|  | N | P-1 | P-2 | N | P-1 | P-2 | N | P-1 | P-2 | N | P-1 | P-2 |
| **Training dataset** | | |  |  |  |  |  |  |  |  |  |  |
| 1–3 cycles | 8 | 0.162 | 0.250 | 54 | 0.006 | 0.009 | 20 | 0.031 | 0.045 | 6 | 0.061 | 0.130 |
| 4–6 cycles | 7 | 0.101 | 0.250 | 34 | 0.001 | 0.005 | 12 | 0.038 | 0.033 | 2 | 0.013 | 0.043 |
| >6 cycles | 4 | Ref | Ref | 38 | Ref | Ref | 12 | Ref | Ref | 4 | Ref | Ref |
| **External validation dataset 1** | | | |  | | |  | | |  | | |
| 1–3 cycles | 1 | 0.47 | 0.47 | 12 | <0.001 | <0.001 | 36 | 0.018 | 0.018 | 28 | 0.381 | 0.380 |
| 4–6 cycles | 1 | 1.00 | 1.00 | 20 | <0.001 | 0.005 | 30 | 0.014 | 0.014 | 17 | 0.388 | 0.380 |
| >6cycles | 2 | Ref | Ref | 12 | Ref | Ref | 21 | Ref | Ref | 7 | Ref | Ref |
| **External validation dataset 2** | | | |  | | |  | | |  | | |
| 1–3 cycles | 1 | 0.076 | 0.076 | 23 | <0.001 | <0.001 | 34 | <0.001 | <0.001 | 13 | 0.009 | 0.001 |
| 4–6 cycles | 3 | 0.994 | 0.994 | 15 | <0.001 | <0.001 | 16 | 0.005 | 0.006 | 6 | 0.355 | 0.172 |
| >6cycles | 5 | Ref | Ref | 28 | Ref | Ref | 20 | Ref | Ref | 13 | Ref | Ref |

**Table S5. Comparative analysis of overall survival across the three subgroups of patients recommended short‑course immunotherapy by OPST.** Both unadjusted p‑values (P-1) and p‑values adjusted using the Benjamini‑Hochberg method (P-2) are presented.

|  | Subgroup 2 | | | Subgroup 4 | | | | Subgroup 6 | | |
| --- | --- | --- | --- | --- | --- | --- | --- | --- | --- | --- |
|  | N | P-1 | P-2 | N | | P-1 | P-2 | N | P-1 | P-2 |
| **Training dataset** | | |  |  | |  |  |  |  |  |
| 1–3 cycles | 10 | Ref | Ref | 32 | | Ref | Ref | 27 | Ref | Ref |
| 4–6 cycles | 10 | 0.501 | 0.830 | 23 | | 0.003 | 0.011 | 20 | 0.003 | 0.012 |
| >6 cycles | 12 | 0.622 | 0.830 | 20 | | 0.009 | 0.015 | 17 | 0.022 | 0.044 |
| **External validation dataset 1** | | | |  | | | |  | | |
| 1–3 cycles | 9 | Ref | Ref | 17 | Ref | | Ref | 25 | Ref | Ref |
| 4–6 cycles | 2 | 0.944 | 0.940 | 13 | 0.029 | | 0.029 | 23 | 0.016 | 0.016 |
| >6 cycles | 4 | 0.103 | 0.100 | 18 | 0.030 | | 0.040 | 26 | 0.030 | 0.040 |
| **External validation dataset 2** | | | |  | | | |  | | |
| 1–3 cycles | 22 | Ref | Ref | 19 | Ref | | Ref | 36 | Ref | Ref |
| 4–6 cycles | 10 | 0.771 | 0.770 | 21 | 0.032 | | 0.040 | 13 | <0.001 | <0.001 |
| >6 cycles | 11 | 0.719 | 0.770 | 30 | 0.015 | | 0.030 | 19 | 0.033 | 0.044 |

**Landmark analysis to address time-dependent treatment bias**

We performed landmark analyses at two clinically relevant time points corresponding to early and intermediate treatment exposure: (i) 3 cycles and (ii) 6 cycles of ICI therapy.

For each landmark analysis, only patients who were alive at the corresponding time point were included, so that all patients had an equal opportunity to receive longer treatment. Overall survival (OS) was then evaluated from the landmark time onward.

At the 3-cycle landmark, patients were categorized according to whether they discontinued treatment after short-course therapy (1–3 cycles) or continued treatment (intermediate- or long-course). The results showed that continuation beyond 3 cycles was not associated with improved OS. Multivariable Cox regression including age, longest tumor diameter, dNLR, and other baseline characteristics showed no significant association between continuation versus discontinuation and OS (HR = 0.860, 95% CI: 0.683–1.080, P = 0.211). This pattern was consistent across datasets (external validation cohort 1: HR = 1.000, 95% CI: 0.781–1.290, P = 0.890; external validation cohort 2: HR = 1.060, 95% CI: 0.819–1.380, P = 0.711). Kaplan–Meier survival curves are shown in Figure S10A to S10C (Continue denotes the patients received intermediate- or long-course immunotherapy).

**Figure S10.** Landmark analyses at 3 and 6 cycles in the training dataset (first column), external validation dataset 1 (second column), and external validation dataset 2 (third column).

At the 6-cycle landmark, among patients who remained alive at this time point, continuation to long-course therapy (>6 cycles) was associated with longer OS than discontinuation after intermediate-course therapy (4–6 cycles). Multivariable Cox analysis showed this association in the overall cohort (HR = 0.564, 95% CI: 0.416–0.766, P < 0.0001), with consistent patterns across the validation cohorts (external validation cohort 1: HR = 0.539, 95% CI: 0.379–0.767, P = 0.0002; external validation cohort 2: HR = 0.450, 95% CI: 0.304–0.668, P = 0.0002). Kaplan–Meier survival curves are shown in Figure S10D to S10F.

**Analysis of immune-related adverse events and discontinuation reasons**

To further evaluate potential confounding factors that may influence treatment duration, we conducted detailed analyses of immune-related adverse events (irAEs) and reasons for treatment discontinuation.

First, for irAEs, we retrospectively reviewed patient-level data to collect detailed information on immune-related toxicity. A total of 173, 118, and 126 patients with clearly documented toxicity grades were identified in the training cohort, external validation cohort 1, and external validation cohort 2, respectively. The remaining patients could not be included because of incomplete records, transfer to other institutions, or privacy-related non-disclosure.

Within each of the seven OPST-defined subgroups, we compared the distribution of irAE grades across patients receiving short-, intermediate-, and long-course therapy. No statistically significant differences were observed across treatment-duration groups within any subgroup in the training cohort (all P > 0.05), and similar findings were observed in both external validation cohorts, with the exception of one subgroup in external validation cohort 2, as shown in Tables S6 to S8.

**Table S6. Distribution of adverse events (G1–G4) among patients receiving short-, intermediate-, and long-course immunotherapy across the seven OPST-defined subgroups in the training dataset, with within-subgroup comparisons between treatment duration and adverse events.** P values were obtained using the Kruskal–Wallis test with Benjamini–Hochberg correction.

|  | G1 | G2 | G3 | G4 | P value |
| --- | --- | --- | --- | --- | --- |
| Subgroup 1 |  |  |  |  | 0.861 |
| Short | 3 | 0 | 1 | 0 |  |
| Intermediate | 2 | 1 | 0 | 0 |  |
| Long | 1 | 0 | 1 | 0 |  |
| Subgroup 2 |  |  |  |  | 0.752 |
| Short | 1 | 2 | 0 | 1 |  |
| Intermediate | 2 | 1 | 0 | 0 |  |
| Long | 4 | 1 | 1 | 0 |  |
| Subgroup 3 |  |  |  |  | 0.398 |
| Short | 17 | 1 | 5 | 0 |  |
| Intermediate | 18 | 2 | 2 | 0 |  |
| Long | 11 | 4 | 3 | 0 |  |
| Subgroup 4 |  |  |  |  | 0.900 |
| Short | 7 | 4 | 1 | 0 |  |
| Intermediate | 7 | 1 | 1 | 0 |  |
| Long | 6 | 3 | 1 | 0 |  |
| Subgroup 5 |  |  |  |  | 0.849 |
| Short | 6 | 3 | 2 | 0 |  |
| Intermediate | 2 | 0 | 1 | 0 |  |
| Long | 6 | 0 | 2 | 0 |  |
| Subgroup 6 |  |  |  |  | 0.365 |
| Short | 9 | 2 | 1 | 0 |  |
| Intermediate | 4 | 1 | 3 | 0 |  |
| Long | 6 | 1 | 0 | 0 |  |
| Subgroup 7 |  |  |  |  | 0.920 |
| Short | 2 | 1 | 1 | 0 |  |
| Intermediate | 0 | 0 | 0 | 0 |  |
| Long | 3 | 0 | 1 | 0 |  |

**Table S7. Distribution of adverse events (G1–G4) among patients receiving short-, intermediate-, and long-course immunotherapy across the seven OPST-defined subgroups in external validation dataset 1, with within-subgroup comparisons between treatment duration and adverse events.** P values were obtained using the Kruskal–Wallis test with Benjamini–Hochberg correction.

|  | G1 | G2 | G3 | G4 | P value |
| --- | --- | --- | --- | --- | --- |
| Subgroup 1 |  |  |  |  | NA |
| Short | 0 | 0 | 0 | 0 |  |
| Intermediate | 0 | 0 | 0 | 0 |  |
| Long | 0 | 0 | 0 | 0 |  |
| Subgroup 2 |  |  |  |  | 0.820 |
| Short | 1 | 0 | 1 | 1 |  |
| Intermediate | 0 | 1 | 0 | 0 |  |
| Long | 2 | 1 | 0 | 0 |  |
| Subgroup 3 |  |  |  |  | 0.696 |
| Short | 0 | 2 | 0 | 0 |  |
| Intermediate | 4 | 2 | 1 | 0 |  |
| Long | 2 | 2 | 1 | 0 |  |
| Subgroup 4 |  |  |  |  | 0.971 |
| Short | 0 | 2 | 1 | 0 |  |
| Intermediate | 1 | 2 | 0 | 0 |  |
| Long | 2 | 4 | 2 | 0 |  |
| Subgroup 5 |  |  |  |  | 0.904 |
| Short | 7 | 5 | 3 | 1 |  |
| Intermediate | 2 | 7 | 1 | 0 |  |
| Long | 4 | 3 | 3 | 0 |  |
| Subgroup 6 |  |  |  |  | 0.253 |
| Short | 1 | 3 | 4 | 0 |  |
| Intermediate | 4 | 2 | 2 | 0 |  |
| Long | 1 | 7 | 2 | 0 |  |
| Subgroup 7 |  |  |  |  | 0.430 |
| Short | 1 | 7 | 2 | 0 |  |
| Intermediate | 2 | 5 | 0 | 0 |  |
| Long | 0 | 3 | 1 | 0 |  |

**Table S8. Distribution of adverse events (G1–G4) among patients receiving short-, intermediate-, and long-course immunotherapy across the seven OPST-defined subgroups in external validation dataset 2, with within-subgroup comparisons between treatment duration and adverse events.** P values were obtained using the Kruskal–Wallis test with Benjamini–Hochberg correction.

|  | G1 | G2 | G3 | G4 | P value |
| --- | --- | --- | --- | --- | --- |
| Subgroup 1 |  |  |  |  | 0.828 |
| Short | 0 | 0 | 0 | 0 |  |
| Intermediate | 0 | 2 | 0 | 0 |  |
| Long | 1 | 2 | 0 | 0 |  |
| Subgroup 2 |  |  |  |  | 0.151 |
| Short | 5 | 2 | 1 | 0 |  |
| Intermediate | 2 | 1 | 0 | 0 |  |
| Long | 0 | 2 | 2 | 0 |  |
| Subgroup 3 |  |  |  |  | 0.002 |
| Short | 3 | 0 | 0 | 0 |  |
| Intermediate | 4 | 1 | 0 | 0 |  |
| Long | 1 | 11 | 0 | 0 |  |
| Subgroup 4 |  |  |  |  | 0.810 |
| Short | 3 | 3 | 0 | 0 |  |
| Intermediate | 2 | 4 | 0 | 0 |  |
| Long | 5 | 7 | 3 | 0 |  |
| Subgroup 5 |  |  |  |  | 0.091 |
| Short | 5 | 1 | 0 | 0 |  |
| Intermediate | 1 | 5 | 1 | 1 |  |
| Long | 7 | 3 | 1 | 0 |  |
| Subgroup 6 |  |  |  |  | 0.327 |
| Short | 4 | 2 | 1 | 1 |  |
| Intermediate | 1 | 1 | 2 | 0 |  |
| Long | 6 | 0 | 1 | 0 |  |
| Subgroup 7 |  |  |  |  | 0.175 |
| Short | 3 | 1 | 0 | 0 |  |
| Intermediate | 0 | 4 | 0 | 0 |  |
| Long | 3 | 3 | 1 | 0 |  |

For treatment discontinuation reasons, we retrieved structured or free-text records of all the included patients and obtained 256, 288, and 231 patients with the corresponding data in the training cohort, external validation cohort 1, and external validation cohort 2, respectively. Reasons for discontinuation in the remaining patients could not be obtained because of privacy constraints, including financial or family-related factors, or incomplete documentation.

Given the heterogeneity of the original records, free-text discontinuation data were systematically reviewed and consolidated into four clinically meaningful categories using a hierarchical classification approach:

irAEs: toxicity requiring treatment discontinuation, including pneumonitis, dermatologic events, cardiac toxicity, or other clinically significant conditions precluding further ICI therapy.

Disease progression (DP): discontinuation due to radiographic or clinical progression.

Treatment completion or conversion (TCC): planned discontinuation after reaching a clinical endpoint, including completion of maintenance therapy, conversion to surgery, transition to radiotherapy, or switch to targeted therapy.

Others: including loss to follow-up, financial constraints, patient or family decision, COVID-19-related interruption, or unclear documentation.

Across the training and external validation cohorts, no statistically significant differences in the distribution of discontinuation reasons across treatment-duration groups were observed within any OPST-defined subgroup (see Tables S9 to S11), with the exception of Subgroup 7 in external validation cohort 2 (n = 23, P = 0.045, Table S11).

**Table S9. Distribution of treatment discontinuation reasons among patients receiving short-, intermediate-, and long-course immunotherapy across the seven OPST-defined subgroups in the training dataset, with within-subgroup comparisons between treatment duration and discontinuation reasons.** P values were obtained using the Kruskal–Wallis test with Benjamini–Hochberg correction. Discrepancies in irAE counts between this table and the G1–G4 statistics in the previous tables reflect the fact that some patients had adverse events without documented grading, whereas in other cases adverse events were recorded but were not the primary reason for treatment discontinuation.

|  | irAEs | DP | TCC | Others | P value |
| --- | --- | --- | --- | --- | --- |
| Subgroup 1 |  |  |  |  | 0.895 |
| Short | 1 | 2 | 0 | 4 |  |
| Intermediate | 0 | 1 | 0 | 5 |  |
| Long | 0 | 1 | 0 | 1 |  |
| Subgroup 2 |  |  |  |  | 0.939 |
| Short | 2 | 3 | 1 | 3 |  |
| Intermediate | 1 | 4 | 1 | 1 |  |
| Long | 0 | 2 | 1 | 2 |  |
| Subgroup 3 |  |  |  |  | 0.479 |
| Short | 3 | 17 | 4 | 11 |  |
| Intermediate | 5 | 15 | 0 | 7 |  |
| Long | 4 | 12 | 1 | 11 |  |
| Subgroup 4 |  |  |  |  | 0.541 |
| Short | 6 | 6 | 1 | 12 |  |
| Intermediate | 0 | 6 | 2 | 6 |  |
| Long | 1 | 7 | 1 | 3 |  |
| Subgroup 5 |  |  |  |  | 0.611 |
| Short | 2 | 5 | 0 | 4 |  |
| Intermediate | 1 | 5 | 1 | 1 |  |
| Long | 0 | 4 | 1 | 4 |  |
| Subgroup 6 |  |  |  |  | 0.455 |
| Short | 3 | 14 | 0 | 3 |  |
| Intermediate | 2 | 7 | 0 | 3 |  |
| Long | 2 | 2 | 1 | 4 |  |
| Subgroup 7 |  |  |  |  | 0.337 |
| Short | 1 | 2 | 0 | 3 |  |
| Intermediate | 0 | 0 | 0 | 2 |  |
| Long | 1 | 1 | 0 | 0 |  |

**Table S10. Distribution of treatment discontinuation reasons among patients receiving short-, intermediate-, and long-course immunotherapy across the seven OPST-defined subgroups in external validation dataset 1, with within-subgroup comparisons between treatment duration and discontinuation reasons.** P values were obtained using the Kruskal–Wallis test with Benjamini–Hochberg correction. Discrepancies in irAE counts between this table and the G1–G4 statistics in the previous tables reflect the fact that some patients had adverse events without documented grading, whereas in other cases adverse events were recorded but were not the primary reason for treatment discontinuation.

|  | irAEs | DP | TCC | Others | P value |
| --- | --- | --- | --- | --- | --- |
| Subgroup 1 |  |  |  |  | 0.735 |
| Short | 0 | 1 | 0 | 0 |  |
| Intermediate | 0 | 0 | 0 | 1 |  |
| Long | 0 | 1 | 0 | 0 |  |
| Subgroup 2 |  |  |  |  | 0.406 |
| Short | 1 | 3 | 0 | 4 |  |
| Intermediate | 1 | 0 | 0 | 0 |  |
| Long | 0 | 1 | 1 | 2 |  |
| Subgroup 3 |  |  |  |  | 0.726 |
| Short | 1 | 4 | 0 | 6 |  |
| Intermediate | 2 | 8 | 2 | 6 |  |
| Long | 0 | 7 | 0 | 5 |  |
| Subgroup 4 |  |  |  |  | 0.990 |
| Short | 1 | 6 | 2 | 4 |  |
| Intermediate | 2 | 5 | 0 | 5 |  |
| Long | 1 | 8 | 1 | 5 |  |
| Subgroup 5 |  |  |  |  | 0.732 |
| Short | 5 | 13 | 3 | 10 |  |
| Intermediate | 7 | 11 | 1 | 7 |  |
| Long | 2 | 12 | 1 | 5 |  |
| Subgroup 6 |  |  |  |  | 0.092 |
| Short | 5 | 1 | 2 | 15 |  |
| Intermediate | 1 | 5 | 2 | 13 |  |
| Long | 2 | 13 | 2 | 8 |  |
| Subgroup 7 |  |  |  |  | 0.240 |
| Short | 6 | 8 | 0 | 11 |  |
| Intermediate | 2 | 9 | 0 | 4 |  |
| Long | 2 | 3 | 0 | 0 |  |

**Table S11. Distribution of treatment discontinuation reasons among patients receiving short-, intermediate-, and long-course immunotherapy across the seven OPST-defined subgroups in external validation dataset 2, with within-subgroup comparisons between treatment duration and discontinuation reasons.** P values were obtained using the Kruskal–Wallis test with Benjamini–Hochberg correction. Discrepancies in irAE counts between this table and the G1–G4 statistics in the previous tables reflect the fact that some patients had adverse events without documented grading, whereas in other cases adverse events were recorded but were not the primary reason for treatment discontinuation.

|  | irAEs | DP | TCC | Others | P value |
| --- | --- | --- | --- | --- | --- |
| Subgroup 1 |  |  |  |  | 1.000 |
| Short | 0 | 0 | 0 | 0 |  |
| Intermediate | 0 | 1 | 0 | 2 |  |
| Long | 1 | 1 | 0 | 3 |  |
| Subgroup 2 |  |  |  |  | 0.903 |
| Short | 1 | 1 | 1 | 10 |  |
| Intermediate | 0 | 2 | 1 | 4 |  |
| Long | 1 | 2 | 1 | 6 |  |
| Subgroup 3 |  |  |  |  | 0.607 |
| Short | 0 | 1 | 0 | 3 |  |
| Intermediate | 0 | 2 | 0 | 10 |  |
| Long | 3 | 5 | 1 | 13 |  |
| Subgroup 4 |  |  |  |  | 0.552 |
| Short | 0 | 4 | 1 | 6 |  |
| Intermediate | 0 | 3 | 0 | 10 |  |
| Long | 0 | 5 | 6 | 18 |  |
| Subgroup 5 |  |  |  |  | 0.983 |
| Short | 2 | 1 | 0 | 6 |  |
| Intermediate | 2 | 2 | 0 | 7 |  |
| Long | 0 | 4 | 1 | 5 |  |
| Subgroup 6 |  |  |  |  | 0.849 |
| Short | 2 | 4 | 2 | 16 |  |
| Intermediate | 2 | 2 | 1 | 4 |  |
| Long | 1 | 3 | 2 | 10 |  |
| Subgroup 7 |  |  |  |  | 0.045* |
| Short | 0 | 0 | 0 | 7 |  |
| Intermediate | 2 | 0 | 1 | 3 |  |
| Long | 0 | 1 | 0 | 9 |  |

**Association of adverse events and discontinuation with survival**

To further explore the clinical relevance of these factors, we additionally conducted Kaplan–Meier analyses stratified by irAE grade (G1–G4) and discontinuation category (irAEs, DP, TCC, and Others).

Across datasets, survival patterns stratified by both discontinuation categories (Figure S11A to S11C) and adverse events (Figure S11D to S11F) were heterogeneous. For example, patients who discontinued treatment because of irAEs showed the most favorable survival in the training cohort, but this pattern was not consistently reproduced in the validation cohorts.

**Figure S11.** Kaplan–Meier survival curves according to irAE grade and discontinuation category in the training dataset (first column), external validation dataset 1 (second column), and external validation dataset 2 (third column). TCC, treatment completion or conversion.

The counterfactual random forest was used as an intermediate policy-learning component to estimate, for each patient, the expected clinical reward under each candidate treatment-duration strategy. Baseline covariates included the variables used for policy learning, and treatment duration was encoded as a multi-arm exposure corresponding to the three duration categories considered in the study (short-, intermediate-, and long-course therapy). The forest was trained to estimate arm-specific conditional outcome functions, that is, the expected survival-related outcome under each treatment-duration option conditional on baseline covariates. This follows the generalized random forest framework for heterogeneous treatment effect estimation and policy learning, and details are presented as below.

First, we define the inputs of random forest, in this study, baseline covariates were defined as $X$(e.g., age, tumor diameter, dNLR), treatment was defined as $A\in\left\{ 1,2,3 \right\}$(short-, intermediate-, long-course), and outcome was defined as $Y$(e.g., overall survival).

Then, we estimate the treatment assignment mechanism by fitting a model to estimate propensity scores:

$$\hat{e}_{a}(X)=P(A=a\mid X)$$

To fit the counterfactual random forest, we then train a generalized random forest to estimate arm-specific outcome functions:

$$\hat{\mu}_{a}(X)=\mathbb{E}[Y\mid X,A=a]$$

For each patient, predicted outcomes under all treatment options (including unobserved ones) were obtained.

Using these estimates, we then constructed a patient-by-treatment reward matrix, in which each row corresponded to one patient and each column to one candidate treatment-duration strategy. The entries of this matrix represented the estimated reward that the patient would receive under that strategy. The reward for patient *i* under treatment option *a* can be calculated as the estimated conditional outcome,

$$\hat{R}_{i,a}=\hat{\mu}_{a}(X_{i}),$$

where $\hat{\mu}_{a}(X_{i})$ denotes the counterfactual outcome estimated by the forest for patient *i* if assigned to treatment duration *a*. When doubly robust policy learning was used, the reward matrix was calculated using doubly robust pseudo-outcomes of the form

$$\hat{R}_{i,a}=\hat{\mu}_{a}(X_{i})+\frac{\mathbb{I}(A_{i}=a)}{\hat{e}_{a}(X_{i})}\left( Y_{i}-\hat{\mu}_{a}(X_{i}) \right),$$

where $A_{i}$ is the observed treatment assignment, $Y_{i}$ is the observed outcome, and $\hat{e}_{a}(X_{i})$ is the estimated propensity for receiving treatment *a*. This construction provides a reward proxy for each treatment arm at the individual level and is the standard input for empirical welfare maximization and policy-tree learning.

The resulting reward-estimation matrix was then supplied to the Optimal Policy Survival Tree procedure, which recursively partitioned the covariate space and selected, within each terminal node, the treatment-duration strategy with the highest estimated mean reward. In this way, the tree was learned not as a prognostic model, but as an interpretable policy model that maps baseline clinical characteristics to the treatment duration estimated to yield the best outcome. This is conceptually aligned with interpretable tree-based policy learning using doubly robust reward estimates.

Based on the above details, we have clarified the covariates and treatment arms used in the counterfactual random forest; the exact outcome/reward definition; the formula used to construct the reward-estimation matrix; and a clearer description of how this matrix was used to fit the OPST. We believe that the revised manuscript has clarified that the counterfactual random forest was used to estimate individualized arm-specific rewards, and that the reward-estimation matrix served as the bridge between counterfactual outcome estimation and interpretable policy-tree construction.
